# The haematological side effects of clozapine: literature review and meta-analysis

**DOI:** 10.1192/bjo.2021.729

**Published:** 2021-06-18

**Authors:** Tariq Munshi, Farooq Naeem, Mohammed Ayub, Saeed Farooq, Davit Khachatryan, Selim Asmer, Peter Wang, Shahista Premji, Christine Van Winssen, Felix Lau, Jonathan Fairbairn

**Affiliations:** 1 St Michael's Hospital, University of Toronto; 2 Canadian Addictions and Mental Health, University of Toronto; 3 Queens University; 4 Keele University; 5 Queen's University

## Abstract

**Aims:**

This review and meta-analysis aim to estimate the cumulative incidence of clozapine induced agranulocytosis and leukopenia the impact of the associated factors such as dose of clozapine, duration of follow-up, gender and race on the cumulative incidence.

**Background:**

Clozapine is the only medication licensed for treatment-resistant schizophrenia. There has been a renewed interest in the role of Clozapine in the treatment of Schizophrenia based on strong evidence that favours its efficacy and safety. Despite the evidence that Clozapine has superior efficacy and has been recommended for treatment-resistant cases by the national guidelines, the drug is underutilised.

**Method:**

We included all studies in which clozapine was used for a psychotic illness. We included studies which provided data on two primary indices; Leucopenia or agranulocytosis and neutropenia; defined according to the cut off used by CPMS for total WBC and neutrophil count. Additionally we included studies reporting another blood dyscrasia or death due to agranulocytosis. Studies were identified by searching AMED, BIOSIS, CINAHL, EMBASE, MEDLINE, PsycINFO, PubMed, and registries of Clinical Trials and their monthly updates, hand searches, gray literature, and conference proceedings from the first available date until 2nd February, 2015. The search was updated on 15th March, 2017. The Protocol was initiated and then registered with PROSPERO International prospective register of systematic reviews University of York, Centre for Reviews and Dissemination.

**Result:**

The cumulative incidence of the agranulocytosis in all studies was 00.32 % (CI 00.1-0.63). The cumulative incidence in all studies for different types of blood dyscrasia were following: leucopenia 00.96 % (CI 0.39-1.70), neutropenia 2.93 % (CI 1.49-4.72), other blood dyscrasias 4.64% (CI 2.34-7.52) and any blood dyscrasia was 2.23 (CI 1.46-3.12).

**Conclusion:**

The limitations of this review are mainly due to the nature of evidence from the included studies. We adopted a broad inclusion criteria to include all the available evidence. Number of patients started on Clozapine may be withdrawn from the Clozapine on the earliest signs of blood dyscrasias since the introduction of Clozapine monitoring services. This means that the true incidence of agranulocytosis and neutropenia may be higher and this may be a major bias in finding the true incidence of Clozapine induced neutropenia.

